# The subjective relevance of perceived sound aspects in remote singing educationa)

**DOI:** 10.1121/10.0009143

**Published:** 2022-01-25

**Authors:** Jan Otčenášek, Marek Frič, Eva Dvořáková, Zdeněk Otčenášek, Sven Ubik

**Affiliations:** 1 Music and Dance Faculty of the Academy of Performing Arts in Prague, Prague, Czech Republic; 2 Cesnet, Prague, Czech Republic

## Abstract

One of the consequences of the pandemic is a transition to remote education and the use of network audiovisual communication tools for education in musical disciplines. The circumstances of such education can differ and might influence the perceived sound or the education. The research observes the ratings of perceived aspects in singing lessons taught in three settings (*common*, *reference*, and *direct*). A variance of several aspects that relate to the perceived sound (temporal qualities and qualities of the sound and room) is observed in the remote forms, suggesting that these can be impaired in some settings and significant in the experience. The findings are discussed in relation to the perceived conditions and present practice.

## I. INTRODUCTION

Producing and perceiving sound is essential in musical interaction. The pandemic has resulted in a shift to remote education using various means of network communication in a range of disciplines that depend on auditory interaction, including musical performing arts or choir singing (Daffern *et al.*, 2021; Parkes, 2010). The remote means had been previously used for home education (Dammers, 2009) or access to a distant specialist (Rottondi *et al.*, 2016; Delle Monache *et al.*, 2019), but the scale of the present use, along with a diversity of the used settings, highlight the topics related to their influence on the perceived sound or the education.

Although transmissions of multichannel or uncompressed audio and video over the network and a high accuracy of reproduction of the perceived environment can be achieved (Paul, 2009; Hammershoi and Moler, 2005), these conditions are not commonly met in the practice of general remote musical lessons due to numerous constraints, including those related to settings of the environment or transmission chain. (1) The *common* services for internet conferencing and general purpose distance collaboration that are both popular and available for broad use, are optimized for low bandwidth and use lossy compression of the audio and video, signal filtration, dynamic range compression, or noise and echo suppression (Chen *et al.*, 2012; Exarchakos *et al.*, 2011; Xue and Lower, 2010). This processing alters the signal and increases the sound delay beyond that which would be typical for sound propagation in live settings. The used devices also commonly feature inappropriate recording and reproduction equipment (Van Renterghem *et al.*, 2011). In contrast, high quality audiovisual network transmissions can also be currently performed using appropriate tools and techniques (Delle Monache *et al.*, 2018; Drioli *et al.*, 2013) and are used for distant musical education in some higher education institutions. Although the equipment and methods present (2) a *reference* in terms of the best current practice and technical parameters of the electroacoustic signal chain, the reproduced sound in this form of education can still be influenced by circumstances such as room coupling or recording and reproduction techniques, that might influence the perceived qualities of the sound source or environment (Boren and Genovese, 2018; Meindl *et al.*, 2006; Kassier *et al.*, 2005). Since previous studies in the field have focused on temporal factors (Delle Monache *et al.*, 2018; Ubik *et al.*, 2020), the influence of the remote context on these other qualities is a subject of interest.

The present study, therefore, focuses on investigating the aspects of subjective experience in lessons in settings of the remote (the *common* and *reference* above) and *direct* forms of singing education. The objectives are to investigate the significance of perceived sound in the remote forms, present the associated qualities, and discuss them in relation to the settings. The significance is assessed in terms of a (1) higher deviance of evaluations from *usual* and (2) higher difference of evaluations in the different remote settings.

## II. METHODS

The analysed data were collected in singing lessons of students of acting at the Academy of Performing Arts in Prague (AMU), in three studies that match the three studied settings. The data on the *direct* and *reference* forms originate in experimental lessons that were part of a larger project aimed at developing techniques for remote education. A total of 21 students (first or third year) split by chance into *reference* (6) and *direct* (15). The data on the *common* form originate in lessons observed in the following semester during the pandemic when the students of *direct* attended the same course in remote settings using common services and devices. The tutors were the same in all lessons.

The *direct* lessons took place in a room used for singing lessons at the AMU campus (7 × 5 × 3 m, RT_60_ 0.7 s). The participants included 12 students (7 female and 5 male) and 2 tutors (all female); 3 students from the initial 15 dropped out before the start. Each student took direct lessons during the semester in a constant tutor pair (random assignment) and then three direct lessons in its final month. The tutor and student completed a questionnaire after each lesson in the final month. The tutor and students conformed to natural positions on both sides of a piano (∼2 m apart).

The *reference* lessons took place in a pair of equally sized rooms at the AMU campus (the room of tutors remained the same as above). The participants included six students (four female and two male) and two tutors (all female). Each student took direct individual lessons in a constant tutor pair (random assignment) during the semester and then three remote lessons in its final month. The tutor and student completed a questionnaire after each remote lesson. The audiovisual reproduction equipment in the room included a Genelec 1030A speaker (1 m and 0° ahead; Lisalmi, Finland) and a Shure 81 microphone (20° and 1 m ahead; Niles, IL), placed in standard mono positions (Ballou, 2008), an LG 7100 screen (Seoul, South Korea), and a Blackmagic Ursa Mini camera (1.5 m and 0° ahead; Blackmagic Design, Melbourne, Australia). A sound engineer (tonmeister) operated the equipment (position, sound level). Both of the rooms (900 m distant) were connected by an audiovisual stream [24/48 kHz lossless pulse-code (PCM) audio], sent over a dedicated optical network (1 Gbit) using a modular audio and video transmission platform (MVTP) streaming device (Cesnet, Prague, Czech Republic). The input-output audio latency in the experiment ranged ∼1–4 ms (measured using the audio signal shift of transient 1 kHz sine bursts; Ubik *et al.,* 2020).

The *common* lessons took place in the homes of the participants, unassisted in their choices, using commonly available services and devices. The participants included 14 students (8 female and 6 male) and 2 tutors (all female). The tutors and students completed a questionnaire after one random lesson in a one-month interval (as per instructions) and reported the services [student, Skype 8 (Luxembourg, Luxembourg), Teams 2 (Microsoft, Inc., Redmond, WA), Messenger 2 (Meta Platforms, Menlo Park, CA), Zoom 1 (Zoom, Inc., San Jose, CA); tutor, the same], devices (student, notebook 10, mobile 4, headphone 7, internal speakers 7; tutor, notebook and headphones), and connections [student-tutor, average (ø) upload 25 mbps, ø download 35 mbps; ITU, 2019]. The measured audio input-output latencies for the most frequent service (Skype) on a local network ranged ∼150–220 ms (measured using the audio signal shift of transient 1 kHz sine bursts). Higher values are expected in the actual education due to the influence of servers and distance on the propagation.

The settings in *common* are diverse but differ from those in *reference* in the parameters of the audio signal chain (transducers, signal treatment, latency) and environment (transducer positions, room acoustics).

All participants took the same 1.5 h lessons. The lessons included repeated tasks on the tone range, intonation, and interpretation, and a tutor-student piano accompaniment (both to simultaneous singing and solo) on an acoustic piano in all forms. The tutor and student ratings present dual perspectives on the same education and are, therefore, presented in isolation. The questionnaire consisted of items collected in a prior training remote session (in settings similar to the *reference* settings) in which participants (18 students and 4 tutors) were asked to list all of the aspects that they *consider relevant to the education*. The aspects that were stated by at least one tutor and one student were used as items in the questionnaire to represent aspects of the education experience alongside a pair of evaluative items of the education quality. The aspects integrated into the item text are presented in Table I and their reference to perceived qualities of the settings is noted. The translated meaning of *audition* matches the meaning of an “*act of hearing, especially: a critical hearing*.”^1^ The questionnaires are identical apart from further items in the *common* questionnaires (service, device, and connection) and absent items in the *direct* (temporal items) and tutor questionnaires (*audition-acc*) that are not meaningful (the temporal effects are unnoticeable and tutors are not accompanied). A verbal-anchored numeric scale anchored relative to *what would be considered as usual (normal) relative to a direct education* was used to rate all of the items [-5 (considerably worse), 0 (usual), 5 (considerably better)].

**TABLE I.** The questionnaire inventory (codes and question text). The items that refer to perceived qualities of the settings are presented in bold. The items are organized by their order in the test.

Item code	Item text
**Audition-qual**	**Audition of all taught sound qualities**
**Audition-self**	**Audition of all qualities of my sound**
**Audition-acc**	**Audition of the accompaniment**
**Room sound**	**Perceived character of the room**
**Sound lag**	**Perceived delay of the sound**
**Synchrony**	**Synchrony of visuals and sound**
**Environment**	**Evaluation of the environment**
**Observation**	**Seeing all aspects the lecture**
**Tuition space**	**Ability to operate in the set area**
Pacing	Subjective tempo of the lecture
Focus	Ability to focus on the lecture
Impression	Overall impression from the lecture
Evaluation	Rating relative to a direct experience

## III. ANALYSIS

**FIG. 1.**
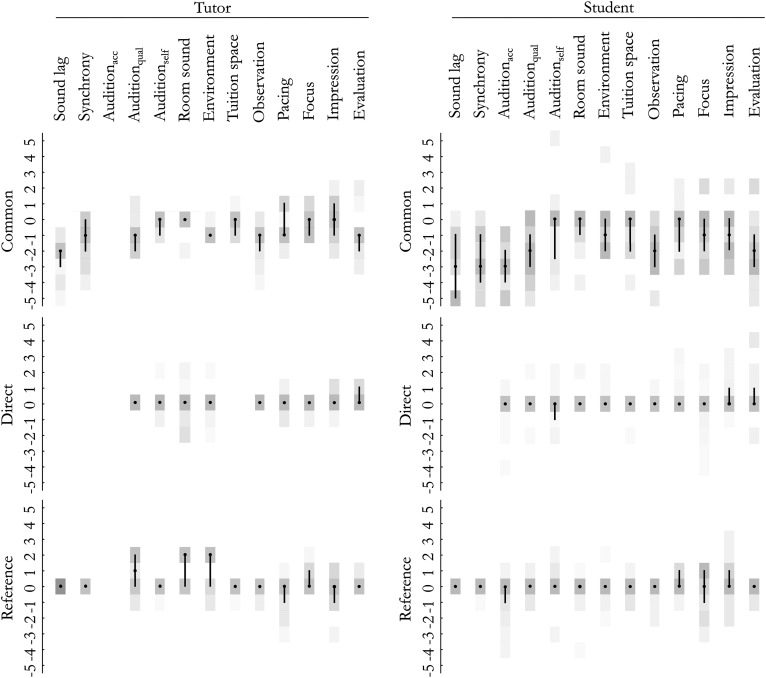
The distributions of the ratings: the medians, interquartile ranges (black), and histograms (grey).

The analysis was conducted in Statistica (2001) and SPSS (IBM, 2010) software. The values in each item are structured into sets (*common, reference, and direct*) and subsets (subject data). The statistics are computed for items in the tutor-student subsets in each set, unless else is stated (available-case method is used to treat missing values). The statistics include the median (*M*), which denotes a median difference from the differential scale midpoint and is used to indicate a magnitude of deviance relative to a *usual* evaluation, and the interquartile range (*Q*_1–3_), which denotes dispersion and is used to indicate deviating evaluations. Both statistics are assessed on each item itself or compared to those of other items or sets, and are presented in the main figure (Fig. 1). A sign-test (two-tails) is used to test the difference of a median parameter (*θ*) of the distribution relative to a zero median (*θ*_0_) parameter. The statistic is used to test the statistical significance of the deviation of the ratings from *usual* (H_0_, *θ* = 0) and confirm that the item evaluation is affected. The items that have significant and higher (>1) median differences in either subset of the remote forms are marked in Tables II and III using solid circles and noted for further discussion. A Kruskall-Wallis (*H*) test and its effect size (*r*) are also computed for each item in both subsets of the *common* and *reference* sets to test for rank differences in the item distributions between the sets and determine their scope (Ellis, 2010). Significant items that have higher *r* (>0.45) are marked and discussed further in relation to the differences between the sets. The *H* and *r* statistics are also computed for gender or year in both subsets of student remote data to assess the influence of confounding variables. A *J-t*_std_ and *r* statistic of a Jonckheere trend test is computed for subsets of the repeated ratings in the students to test for a trend in the repeated ratings. An additional Cronbach *α* is computed for all of the cases in each set to determine the consistency of the ratings of raters.

**TABLE II.** The results of the sign-test: the median (*M*), *p*-value (*p*), and significance (sig.). The solid circles mark items with *M* > 1 and *p* < 0.05. *p < 0.05, **p < 0.01, and ***p < 0.001.

	Common						Reference						Direct					
	Tutor			Student			Tutor			Student			Tutor			Student		
	*M*	*p*	Sig.	*M*	*p*	Sig.	*M*	*p*	Sig.	*M*	*p*	Sig.	*M*	*p*	Sig.	*M*	*p*	Sig.
• Sound lag	−2	0.00	***	−3	0.00	***	0	1.0	—	0	1.0	—	—	—		—	—	
• Synchrony	−1	0.00	***	−3	0.00	***	0	1.0	—	0	1.0	—	—	—		—	—	
• Audition_qual_	−1	0.00	***	−2	0.00	***	1	0.07	—	0	1.0	—	0	1.0	—	0	0.69	—
° Audition_self_	0	0.06	—	0	0.45	—	0	1.0	—	0	0.38	—	0	0.73	—	0	0.50	—
• Audition_acc_	—	—		−3	0.00	***	—	—		0	0.07	—	—	—		0	1.0	—
• Room sound	0	0.50	—	0	0.03	*	2	0.00	***	0	0.62	—	0	0.04	*	0	1.0	—
• Environment	−1	0.00	***	−1	0.04	*	2	0.15	—	0	0.63	—	0	0.45	—	0	0.22	—
° Tuition space	0	0.13	—	0	0.29	—	0	1.0	—	0	0.38	—	—	—	—	0	1.0	—
• Observation	−1	0.00	***	−2	0.00	***	0	0.25	—	0	0.13	—	0	1.0	—	0	0.25	—
° Pacing	−1	0.33	—	0	0.18	—	0	0.07	—	0	0.22	—	0	0.75	—	0	0.07	—
° Focus	0	0.54	—	−1	0.11	—	0	0.02	—	0	1.0	—	0	0.38	—	0	0.51	—
• Impression	0	1.0	—	−1	0.02	*	0	1.0	—	0	0.75	—	0	0.07	—	0	0.02	*
• Evaluation	−1	0.01	**	−2	0.02	*	0	1.0	—	0	1.0	—	0	0.13	—	0	0.13	—

**TABLE III.** The Kruskall-Wallis test of the differences of *common* and *reference* or subsets of other variables: the differences of *H*-statistic (*H*) effect size (r) and significance (sig.). The items with *r* > 0.45 are marked with solid circles. *p < 0.05, **p < 0.01, and ***p < 0.001.

	Form (c*ommon* / *reference*)						Rep. trend			Gender			Year		
	Tutor			Students			Student			Student			Student		
	*H*	*r*	Sig.	*H*	*r*	Sig.	*J-t_std_*	*r*	Sig	*H*	*r*	Sig.	*H*	*r*	Sig.
• Sound lag	29.6	0.92	***	24.1	0.88	***	1.3	0.31	—	1.5	−0.22	—	0.7	0.15	—
• Synchrony	17.8	0.71	***	22.3	0.85	***	0.0	0.00	—	0.6	−0.14	—	0.9	0.17	—
• Audition_qual_	15.7	0.67	—	14.2	0.68	—	1.1	0.27	—	3.5	−0.34	—	0.0	0.00	—
° Audition_self_	2.6	0.28	—	2.6	0.29	—	−0.3	−0.08	—	0.0	0.02	—	1.4	−0.21	—
• Audition_acc_	—	—	—	12.1	0.64	***	0.6	0.14	—	0.3	−0.09	—	0.0	0.03	—
• Room sound	13.1	0.61	***	2.6	0.29	—	−0.4	−0.09	—	0.0	−0.01	—	0.0	−0.03	—
• Environment	17.3	0.70	***	4.2	0.37	*	1.3	0.32	—	2.8	−0.30	—	0.1	−0.04	—
° Tuition space	2.4	0.26	—	0.9	0.16	—	−0.5	−0.12	—	6.4	−0.45	**	3.0	0.31	—
• Observation	15.5	0.66	***	13	0.65	***	−0.1	−0.03	—	1.4	−0.21	—	1.9	0.25	—
° Pacing	5.6	0.40	*	1.6	0.23	—	0.8	0.20	—	0.0	0.02	—	0.0	0.04	—
° Focus	0.2	0.26	—	5.3	0.40	*	1.4	0.34	—	0.3	−0.10	—	0.2	−0.09	—
• Impression	0.1	0.05	—	6.7	0.45	**	0.7	0.18	—	0.1	−0.05	—	0.0	0.03	—
• Evaluation	5.2	0.49	*	3.9	0.42	*	−0.5	−0.22	—	0.1	−0.06	—	1.7	0.30	—

## IV. RESULTS

An overview of the descriptive statistics is presented in Fig. 1 and the results of the sign-test are presented in Table II (for each item in the tutor and student subset in all sets). The medians of multiple items in both remote sets (*common* and *reference*) differ from the scale midpoint, indicating that their evaluations deviate from usual. The medians and dispersions are negative on numerous items in both subsets of the *common* set. The median differences are notable and span a higher range of the scale on the items of *sound lag*, *synchrony*, *audition-qual*, and *audition-acc* and also on *observation* and *evaluation*. Several of those items refer to the perceived sound. The medians in the *reference* set are centred around the midpoint on most items, excluding *room sound* (tutors), but are more dispersed relative to *direct*. All of the items that have higher (>1) medians and significant differences of the median parameters are marked in solid circles in Table II.

Table III presents the results of the Kruskall-Wallis test (the statistic and effect size) of a rank difference between the *common* and *reference*. The statistics are the highest on the items of *sound lag*, *synchrony*, *audition*, and *room sound* (and also *observation*), indicating that their distributions are the most different. The effects in both subsets are the highest for *sound lag* and *synchrony* but are also high for *audition-qual*, *audition-acc*, and *room sound*. A difference can also be observed on the evaluative items (*impression*, *evaluation*), but the effects are not as high. The items with higher differences (*r* > 0.45) are marked in solid circles for further discussion. Table III also presents the results of the confounding influence tests and trend test. The trend in the repeated ratings (rep. trend) of the students and the rank difference between the gender or year subsets are minor (*r* < 0.34) on the items of interest. The Cronbach *α* for the student raters is 0.82 in *direct*, 0.72 in *common*, and 0.80 in *reference*, and indicates a high degree of consistency of the ratings in each set.

## V. DISCUSSION

The items that pass the assumptions of a higher evaluation deviance are marked using solid circles in Table II and those that pass the assumption of a difference are marked using solid circles in Table III. The deviances are the most notable in the *common* form. The highest deviances occur on items that refer (*sound delay, audiovisual synchrony, character of the room, audition of sound qualities*) to the perceived sound and its temporal (*sound lag* and *synchrony*) and spatial qualities (*room sound*), or sound qualities (*audition-qual* and *audition-acc*) including those of the piano or voice. Their ratings also differ between the *common* and *reference* forms. Their settings differ in factors of the acoustic environment (*virtual* or *physical* acoustics; Boren and Genovese, 2018; Koskinen *et al.*, 2010) and tonmeister techniques (transducer placement; Meindl *et al.*, 2006) or the parameters of the signal chain (transducers and components, signal processing, and their electroacoustic response; Van Renterghem *et al.*, 2011; Dobrucki, 2011) that have been linked to audible effects in previous studies, and can be discussed in relation to the observed variance (Meindl *et al.*, 2006; Moore and Tan, 2003; Tervo, 2014; Delle Monache *et al.*, 2018). The significance of the temporal aspects matches the latency ranges of the used equipment that have been studied as the most relevant factor of remote musical interaction in multiple studies (Delle Monache *et al.*, 2018; Chew *et al.*, 2005). But a similar deviance of the aspects related to the other qualities of the perceived sound (*audition* and *room sound*) also suggests that these can be of similar importance. This corresponds to their relevance in the context of musical sound (Stanley *et al.*, 2002) and singing voice assessment (Oates *et al.*, 2006) or sensory realism or presence (Witmer and Singer, 1998; Bovik, 2013). Although *room sound* is significant, the results differ in the subsets, suggesting that the perceived spatial factors either do not deviate in all situations or are perceived as other qualities (Koskinen *et al.*, 2010; Luizard *et al.*, 2020).

The evaluations of the auditory aspects are negative and have negative dispersion in the lessons that used the *common* devices and services and are centred around or above *usual* in the *reference* conditions. This suggests that the aspects can be impaired in some settings, and also that the settings in *reference* are adequate in the context of the education (most ratings are in the *usual* range), and the resulting experience might approach that of a direct education (or even exceed it). The above results and a relation of the aspects to critical listening (Tervo *et al.*, 2014) or *spatial* and *timbral* fidelity (Rumsey *et al.*, 2005) also suggest that improving the audible parameters of the signal chain (in addition to its latency) and using tonmeister techniques for mediation (a standard accent placement of a cardioid microphone is used in *reference*) should be beneficial.

## VI. CONCLUSION

These findings expand the assumptions on the relevance of perceived sound in settings similar to the studied reference education (Delle Monache *et al.*, 2019) and indicate prospective topics of further research. This might concern the acoustic attributes and metrics of the qualities (Gupta *et al.*, 2017; Zahorik, 2009), signal chain parameters (Dobrucki, 2011), perceived effects of the *virtual* or *physical* acoustics (Boren and Genovese, 2018; Koskinen *et al.*, 2010), or audio codecs (Valin *et al.*, 2013). But the main aim of the article is to aid the current forms of remote musical education by raising awareness about the relevance of the perceived sound in the experience and presenting that its improvement can be achieved in some settings (such as optimised electroacoustic response) and using standard tonmeister techniques. This includes the settings used here as a reference. The conditions and their improvement might, therefore, be of interest to students, tutors, or the involved institutions, and their attributes present topics of further research.

### A. Limitations of the study

The limitations concern the design, small sample size, uneven repeated ratings, and pandemic context of the study. Although the conclusions are based on combined evidence amid sets and raters and populations (tutors and students), their validity might be limited to a subset of settings and subjects and invites further studies (especially the tutor results cannot be interpreted in isolation). Although the tests indicate a rater consistency (*α*) and lack of a notable trend in repeated observations (*r_J_*_-__*t*_), the validity is limited to the evaluated lessons rather than subjects (except for students in the *common* form).

### B. Summary

The study examines the significance of perceived sound in remote education settings. The results indicate that both the temporal and other qualities of the perceived sound present relevant aspects of the remote experience, and that these can be impaired in some present settings and improved in others.

## Footnotes

^1^
See https://www.merriam-webster.com/dictionary/audition (Last viewed 1/7/2021).
